# Sociogeographic Variation in the Effects of Heat and Cold on Daily Mortality in Japan

**DOI:** 10.2188/jea.JE20130051

**Published:** 2014-01-05

**Authors:** Chris Fook Sheng Ng, Kayo Ueda, Ayano Takeuchi, Hiroshi Nitta, Shoko Konishi, Rinako Bagrowicz, Chiho Watanabe, Akinori Takami

**Affiliations:** 1 Environmental Epidemiology Section, Center for Environmental Health Sciences, National Institute for Environmental Studies, Tsukuba, Ibaraki, Japan; 1 国立環境研究所 環境健康研究センター 環境疫学研究室; 2 Department of Human Ecology, Graduate School of Medicine, The University of Tokyo, Tokyo, Japan; 2 東京大学大学院医学系研究科 人類生態学; 3 Department of Anthropology, University of Washington, Seattle, WA, USA; 3 ワシントン大学 人類学; 4 Center for Regional Environmental Research, National Institute for Environmental Studies, Tsukuba, Ibaraki, Japan; 4 国立環境研究所 地域環境研究センター

**Keywords:** heat, cold, mortality, time-series, distributed lag

## Abstract

**Background:**

Ambient temperature affects mortality in susceptible populations, but regional differences in this association remain unclear in Japan. We conducted a time-series study to examine the variation in the effects of ambient temperature on daily mortality across Japan.

**Methods:**

A total of 731 558 all-age non-accidental deaths in 6 cities during 2002–2007 were analyzed. The association between daily mortality and ambient temperature was examined using distributed lag nonlinear models with Poisson distribution. City-specific estimates were combined using random-effects meta-analysis. Bivariate random-effects meta-regressions were used to examine the moderating effect of city characteristics.

**Results:**

The effect of heat generally persisted for 1 to 2 days. In warmer communities, the effect of cold weather lasted for approximately 1 week. The combined increases in mortality risk due to heat (99th vs 90th percentile of city-specific temperature) and cold (first vs 10th percentile) were 2.21% (95% CI, 1.38%–3.04%) and 3.47% (1.75%–5.21%), respectively. City-specific effects based on absolute temperature changes were more heterogeneous than estimates based on relative changes, which suggests some degree of acclimatization. Northern populations with a cool climate appeared acclimatized to low temperature but were still vulnerable to extreme cold weather. Population density, average income, cost of property rental, and number of nurses appeared to influence variation in heat effect across cities.

**Conclusions:**

We noted clear regional variation in temperature-related increases in mortality risk, which should be considered when planning preventive measures.

## Abstract (ja)

**背景:**

外気温が感受性の高い集団の死亡に影響するということはよく知られているが，日本においてその関連の地域差は明らかにされていない．本研究では外気温と日死亡に対する影響の日本国内での変動を評価するための時系列解析を実施した．

**手法:**

2002年から2007年までの日本の6都市での死亡データ（事故死を除く）計731,558件を解析対象とした．外気温と日死亡との関連は，結果変数である日死亡数にポアソン分布を仮定した非線形分布ラグモデルで解析した．都市ごとに推定された結果は，都市を変量効果としたメタアナリシスで併合した．また，都市ごとの共変量は，都市を変量効果とした二変量メタ回帰によって調整した．

**結果:**

高気温の影響は1～2日間継続し，暖かい地域での低気温の影響は1週間程度継続することが分かった．高気温によって上昇した併合死亡リスク(外気温99％点でのリスクを90％点でのリスクと比較して)は2.21％増で95％信頼区間は1.38-3.04％，低気温によって上昇した併合死亡リスク(外気温1％点でのリスクを10％点でのリスクと比較して)は3.47％増で95％信頼区間は1.75-5.21％であった．都市ごとの日死亡への影響は，外気温の絶対変化の影響の方が相対変化の影響よりばらつきが大きく，ある程度順応の影響が含まれている可能性が示された．涼しい気候の北部地域では低気温への順応がみられるが，極端に低い気温に対しては脆弱であることも示唆された．また，人口密度，平均収入，不動産賃貸料，看護師の数が都市間の外気温影響の変動に影響していることが示唆された．

**結論:**

外気温関連死亡リスクの上昇には明らかな地域間変動が存在した．予防策を講じる場合には，地域間変動に影響する要因を検討する必要がある．

## INTRODUCTION

Exposure to extreme temperatures increases human mortality among vulnerable populations in differing climates.^1^ Because of adaptation to local climate, the magnitude and characteristics of this temperature–mortality association tend to vary by community.^2^^–^^4^ Some studies found a greater cold effect in warmer cities, others reported a greater heat effect in cool regions, and still others have reported both.^3^^,^^5^^,^^6^ Effects of temperature also differ in relation to demographic and socioeconomic factors.^1^^,^^7^ Poor people,^8^ elderly adults,^9^ individuals with pre-existing medical conditions,^10^^,^^11^ and individuals living in large cities^12^ are particularly vulnerable. In large countries with varying climates, understanding geographic and social variation in the health effects of temperature is crucial for identifying susceptible populations and developing preventive measures.

Japan is an island nation that encompasses a broad range of latitudes. Climate varies from cool and temperate in the north to warm and humid in the south. Climate also varies between the east and west coasts, due to seasonal airflows. Current strategies to alleviate temperature-related health impacts are largely related to heat and include early warning systems, educational programs to promote awareness and prevention of heat-related disorders, long-term urban planning, and landscape improvement to reduce heat exposure.^13^ Despite these efforts, a marked increase in heat-related mortality during the last decade has garnered much concern.^14^^,^^15^ To protect vulnerable individuals from the harmful effects of severe weather conditions, it is important to understand how different climates affect various segments of the population in this era of rising global temperature and increasingly unstable climate.^16^^,^^17^

Studies of the adverse health effects of ambient temperature in Japan have been mostly related to heat.^14^^,^^15^^,^^18^^–^^20^ The association between winter temperatures and daily mortality remains unexplored.^21^ The delayed effects of heat and cold are also unknown. Socioeconomic and geographic variations in the temperature-related risk of mortality remain unclear in regions with differing climates. This study assessed variation in heat, cold, and their delayed effects on daily non-accidental mortality in cities around Japan.

## METHODS

### Study location and data

Six cities, from north to southwest Japan, were studied: Sapporo, Sendai, Tokyo, Nagoya, Osaka, and Fukuoka. Together they include a total population of about 21.7 million. The cities were selected based on the availability of monitoring data, size, and climate.

Data on daily mortality in each city were obtained from the Ministry of Health, Labor and Welfare of Japan for the 6-year period 2002–2007. The data on total mortality encompassed all age groups but excluded nonresidents and deaths from injuries and external causes (International Classification of Disease, 10th Revision code A00–R99). Data on weekly flu incidence were obtained from the Japan National Institute of Infectious Diseases and were used to adjust for confounding by flu epidemics during cold seasons. Mean number of daily cases was computed by dividing the weekly total by the number of workdays in a specific week.

Hourly measurements of ambient temperature and relative humidity in each city were acquired from the Japan Meteorological Agency and were used to compute daily 24-hour means. We adjusted for the intermediate effects of particulate matter with an aerodynamic diameter of less than 2.5 µm (PM_2.5_) and ozone (O_3_), based on our previous findings.^22^^–^^24^ The Ministry of the Environment provided data on hourly measurements of fine particles and photochemical oxidants consisting primarily of O_3_ for the 6-year study period. Daily maximum 8-hour moving averages were computed for O_3_, and the 24-hour mean was calculated for PM_2.5_. Within the study period of 2191 days, the proportion of days with missing PM_2.5_ data ranged from 0.4% to 4.2%. There were no missing data on temperature or O_3_.

Cross-sectional data on prevalence of air conditioners (AC) (2004),^25^ population density (2005),^26^^,^^27^ property rental cost (2003),^28^ and unemployment rate (2005)^29^ were available from the Statistics Bureau of the Ministry of Internal Affairs and Communications. Variables except cost of rental were obtained at the city level. Prefectural income per capita (2002) was acquired from the Economic and Social Research Institute.^30^ Data on the number of registered nurses, by prefecture (2008), were provided by the Japanese Nursing Association.^31^

### Statistical analysis

A generalized linear model with a quasi-likelihood approach was used to estimate the effects of temperature and its lags on -total mortality in each city. Daily mortality was assumed to follow an overdispersed Poisson distribution. We started with a basic model of daily mortality without temperature or air pollutant variables. To account for seasonality and longer time trends in the mortality time-series, we used natural cubic splines (NS) of calendar time with 7 degrees of freedom (*df*) per year and knots at quantiles. This method allowed us to capture about 2 months of temporal information in the estimation process.^32^^,^^33^ Day-of-the-week effect was adjusted for using an indicator variable. Humidity was not included because its effect was found to be insignificant in preliminary analysis.^22^^,^^32^ Flu epidemics were controlled for using an indicator for days when the mean daily number of flu cases exceeded the 90th percentile in a given city during the 6-year period.

To model the effect of temperature exposure, we applied a distributed lag nonlinear model developed by Armstrong^34^ and Gasparrini et al.^35^ This method utilizes a cross-basis function, ie, a bidimensional functional space to simultaneously express the relationships of temperature and their lagged exposures with total mortality.^34^^,^^35^ It allows for modeling of a nonlinear exposure–outcome association including the lag dimension. Cumulative risk of exposure including lagged effects can also be computed. The Poisson regression model for the daily number of total deaths in each city was specified as

$$\begin{array}{l} \log[\text{E}({\text{Y}}_{t})]=\alpha +\text{NS}(t,7)+\gamma \text{DOW}+\lambda {\text{FLU}}_{t}\\ +\beta {\text{T}}_{t,l}+\tau {\bar{\text{PM}}}_{t}+\upsilon {\bar{\text{OZ}}}_{t} \end{array}$$ (1)

where E(Y*_t_*) denotes the expected number of deaths on day *t*, *α* denotes the intercept, NS(*t*,7) is a natural cubic spline of time with 7 *df* per year (to adjust for seasonal and longer time component in the mortality time-series), DOW is an indicator variable for day of the week, FLU*_t_* is a dichotomous variable used to control for flu epidemics, T*_t_*_,_*_l_* is a matrix obtained after applying the distributed lag nonlinear model to daily mean temperature with *l* as the number of lag days and *β* as the corresponding vector of coefficients, and ${\bar{\text{PM}}}_{t}$ and ${\bar{\text{OZ}}}_{t}$ are defined as the average of the current and past 2 days’ concentrations of PM_2.5_ and O_3_, respectively (ie, ${\bar{\text{PM}}}_{t}=\frac{1}{3}\sum_{l=0}^{2}{\text{PM}}_{t-l};\;{\bar{\text{OZ}}}_{t}=\frac{1}{3}\sum_{l=0}^{2}{\text{OZ}}_{t-l}$). The choice of 3 days is supported by the findings of previous research.^22^^–^^24^ This method of averaging helps avoid bias related to selecting the most significant lag.^36^

The effect of temperature on day *t* was modeled using the term *β*T*_t_*_,_*_l_*. We used NS with 3 *df* to specify the nonlinear relationship between daily temperature and number of deaths. This decision was based on sensitivity analysis that showed the use of greater than 3 *df* led to an artificial increase in the estimated current-day effect of heat. After fixing the *df* for the spline of temperature, we then determined the *df* for the spline of lagged effects by minimizing the sum of the absolute value of the partial autocorrelation function of residuals. We used 5 *df* for the spline of lag terms.^37^

To better understand the structure of temperature lag in each city, the relative risk (RR) of mortality was computed for lagged temperature exposures up to 20 days. Initial exploratory analysis showed a 3-week period was sufficient for observing the attenuation of delayed effects. RRs for each lag were computed with reference to the 80th percentile temperature in each city. This temperature reference was based on the understanding of the “optimum” temperature, ie, the temperature at which the mortality rate was lowest, as previously described.^20^ For heat, mortality risk was compared between the maximum temperature and 80th percentile temperature in each city. For cold, minimum temperature and 80th percentile temperature were compared. In addition to estimating the RR at each lag, an overall exposure–response curve, including any delayed effects, was plotted for each city.

To understand geographic variation in the temperature–mortality association, cumulative effects based on relative and absolute changes in temperature in each city were computed. Comparison between relative and absolute measures can provide insights into acclimatization to weather in different communities. The rationale for such comparison has been discussed in detail elsewhere.^38^ Briefly, with a high degree of acclimatization, one can expect results to be similar across cities for relative effects and different for absolute effects. To compute the relative effect of heat, we compared mortality risk at the 99th and 90th percentiles of city-specific temperature distribution. For the relative effect of cold, mortality risk at the first percentile was compared to that at the 10th percentile of city-specific temperature. To estimate the effects based on absolute changes in temperature, fixed temperature differences were set for all locations. To determine the absolute effect of heat, we compared mortality risk at 29°C (95th percentile temperature in all cities) with that at 23.5°C (80th percentile). To determine the absolute effect of cold, mortality risk at 0°C (5th percentile) was compared with that at 23.5°C for all cities. These percentile and temperature cut-offs were previously used to capture possible estimates of heat and cold effects from nonlinear exposure–response curves.^6^^,^^38^^–^^40^

To estimate city-specific RRs, the calculations were based on the relative percentile temperature cutoffs at each location, as described earlier. Because the length of temperature lag differs by city, according to high and low temperatures, we used different approaches to select lag duration. The first approach assumed similar delayed effects and assigned a fixed lag duration for all cities (ie, city variation was ignored). The second approach assigned city-specific lags according to the dampening of temperature effects in each city. This method allowed us to understand how selection of city-specific lags affects pooled multicity estimates.

To obtain multicity estimates, city-specific estimates were combined using random-effects meta-analysis with restricted maximum likelihood. Combined estimates were computed using a weighted regression model with the inverse of within- and between-cities variances as weight.^2^ *I*^2^ was computed to quantify the degree of heterogeneity in city effects. To assess the potential influence of latitude and certain socioeconomic factors on the effects of temperature across different cities, bivariate random-effects meta-regression was used to regress the estimated city effects (on natural logarithm) on each city characteristic.^41^^–^^43^ Covariates for latitude and city characteristics were introduced one at a time in the meta-regression model to determine if they could explain heterogeneity in the city-specific effect estimates.

We tested the robustness of effect estimates against different *df* for the smoothing of temperature variable and lag terms. The sensitivity of combined estimates to temperature lags up to 21 days was also assessed. Effect estimates were reported as RR or percentage change [(RR − 1) × 100%] in daily mortality.

All analyses were performed in R (version 2.14.2; R Project for Statistical Computing, Vienna, Austria) utilizing package “dlnm” 1.6.3,^35^ with the significance level defined as 5%, unless otherwise noted.

## RESULTS

The 6 selected cities are spread across Japan and cover about 10° of latitude, from north to southwest Japan (Table 1). Together the cities encompass approximately 21.7 million residents, and a total of 731 558 non-accidental deaths occurred during the 6-year period. Mean mortality ranged from 15.5 to 163.2 cases per day. Most (81.7%) deaths were among individuals older than 64 years; only 0.5% of deaths were among people younger than 20 years. The prevalence of heat-pump AC was lower in Sapporo and Sendai in the north. In addition, fine particulate pollution was lower in the north, whereas O_3_ level was comparable across cities (Table 2).

**Table 1.** Characteristics of study location, total mortality, and prevalence of air conditioners

City	Latitude	Population 2005 (’000)	Daily total mortality				Prevalence of air conditioners^a^ (%)

			Total	Age >64 years (%)	Mean	(Min, Max)	
Sapporo	43° 4′N	1882	70 437	80.8	32.1	(15, 57)	15.9
Sendai	38° 16′N	1025	34 025	81.6	15.5	(4, 33)	76.5
Tokyo	35° 41′N	12 577	357 480	82.1	163.2	(108, 247)	94.4
Nagoya	35° 10′N	2215	93 415	82.4	42.6	(21, 73)	98.4
Osaka	34° 41′N	2629	128 726	81.2	58.8	(31, 94)	97.6
Fukuoka	33° 35′N	1401	47 475	81.1	21.7	(6, 44)	95.2

^a^For households with ≥2 members, based on the 2004 National Survey of Family Income and Expenditure, Statistics Bureau of Japan.

**Table 2.** Summary statistics for daily temperature, fine particulate matter, and ozone concentrations, by city

City	Daily mean temperature (°C)					Daily mean PM_2.5_ (µg/m^3^)		Daily maximum 8-hr O_3_ (ppbv)	
		
	P99	P90	P50	P10	P1	P50	IQR	P50	IQR
Sapporo	25.7	21.0	9.8	−3.3	−6.7	10.9	(8.3, 14.6)	30.8	(26.0, 32.5)
Sendai	27.5	23.8	13.0	1.9	−1.1	11.3	(8.2, 16.6)	36.6	(30.0, 44.5)
Tokyo	30.6	27.5	17.2	6.3	3.6	18.0	(12.5, 24.3)	33.6	(23.4, 36.8)
Nagoya	30.3	27.5	16.7	4.7	1.5	17.9	(12.2, 25.6)	34.3	(24.9, 35.7)
Osaka	30.8	28.8	17.7	6.2	3.2	18.5	(12.9, 25.9)	37.6	(28.9, 49.5)
Fukuoka	30.5	28.2	17.9	6.8	3.1	19.1	(13.4, 27.5)	38.3	(30.3, 47.2)

PM_2.5_, particulate matter with an aerodynamic diameter of <2.5 µm; P99, 99th percentile; P90, 90th percentile; P25, 25th percentile; P10, 10th percentile; P1, 1st percentile; IQR, interquartile range.

**Figure 1.**
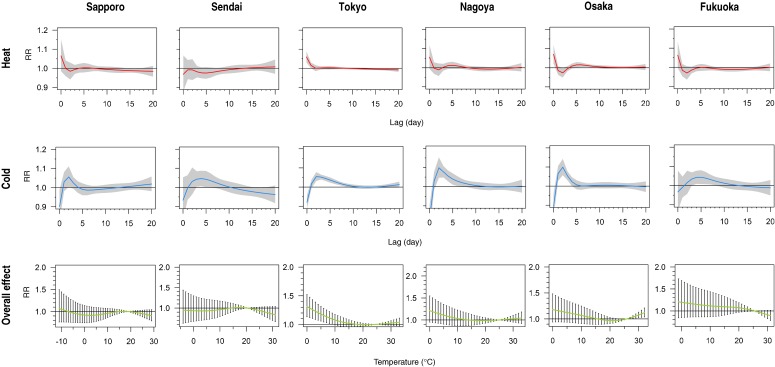
Structure of the delayed effects of heat and cold and the overall effect of temperature on daily mortality, by city. The first and second rows show the relative risks (RRs) of mortality due to heat and cold, from lag 0 to 20. These RR estimates are computed at city-specific maximum (heat) and minimum (cold) temperatures, with the 80th percentile temperature in each city as reference. Shaded regions represent 95% CIs. The third row shows the RR of mortality for the temperature range in each city. The 95% CIs are shown as vertical lines. The degrees of freedom for the natural cubic splines of temperature variable and its lag term are 3 and 5, respectively.

The first and second rows of Figure 1 show the structures of the delayed effects of heat and cold in each city. Heat had the strongest effect on the same day (lag 0) in all cities except Sendai. In Tokyo, heat appeared to have a delayed effect lasting 1 day (lag 1). A tendency of mortality harvesting by heat for 2 to 3 days was discernible in Osaka and Fukuoka, as indicated by the sharp dip below unity RR after lag 0 and the subsequent return to 1. The effect of cold was slower and developed over a longer exposure timeframe. An increase in mortality risk due to cold weather was apparent in warmer cities, especially Tokyo, Nagoya, and Osaka, as evidenced by the significant lags up to approximately day 5. Increasing the *df* for the NS of the lag term beyond 5 did not substantially alter the lag structure (not shown).

Regarding the overall cumulative effects of temperature (Figure 1, third row), Sapporo and Sendai (in the north) had a rather similar monotonous exposure–response relationship. A positive effect of heat was observed in the 3 largest cities: Tokyo, Nagoya, and Osaka. These cities, as well as Fukuoka (in the southwest), showed increasing risk as temperature decreased.

**Figure 2.**
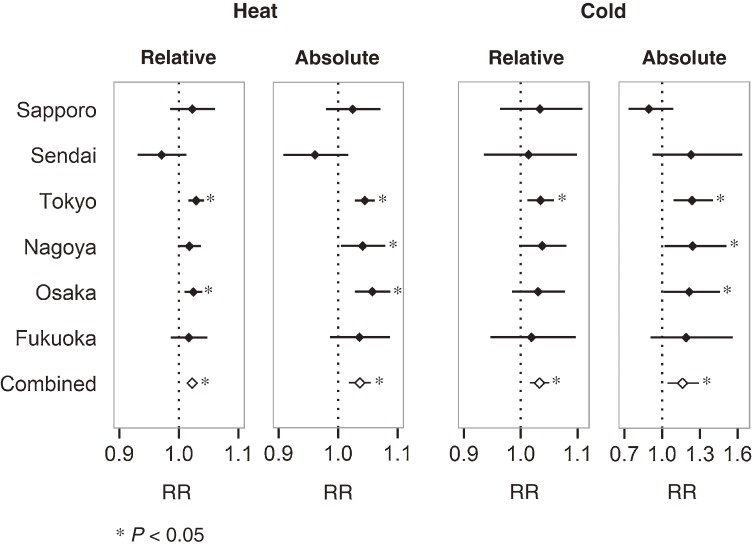
Estimates of relative risk (RR) of heat and cold according to relative and absolute changes in temperature. Estimates of RR according to relative change in temperature were computed by comparing mortality risks at the 99th and 90th percentile for heat and the 1st and 10th percentile for cold. Estimates of RR according to absolute changes in temperature for heat and cold were computed by comparing mortality risks at 29°C to 23.5°C and 0°C to 23.5°C, respectively.

Figure 2 illustrates the estimated effects of temperature, based on relative and absolute changes in temperature. Heat had a positive effect in all cities except Sendai. Tokyo, Nagoya, and Osaka showed a strong heat effect. The combined RR for the effect of heat was 1.02 (95% CI, 1.01–1.03) and 1.04 (95% CI, 1.02–1.05) for relative and absolute temperature changes, respectively. With the exception of Sapporo, the effects of cold were positive in all cities irrespective of whether temperature changes were assessed in relative or absolute terms. In Sapporo, a negative effect was observed when temperature changed in absolute terms (23.5°C vs 0°C). The combined RRs for the relative and absolute effects of cold were 1.03 (95% CI, 1.02–1.05) and 1.16 (95% CI, 1.04–1.29), respectively. Temperature effects based on absolute changes had larger *I*^2^ values (48% and 42% for absolute heat and cold effects, respectively, vs 29% and 0% for relative heat and cold effects), which suggests slightly greater between-city variation in mortality risk estimates for the same temperature difference.

**Figure 3.**
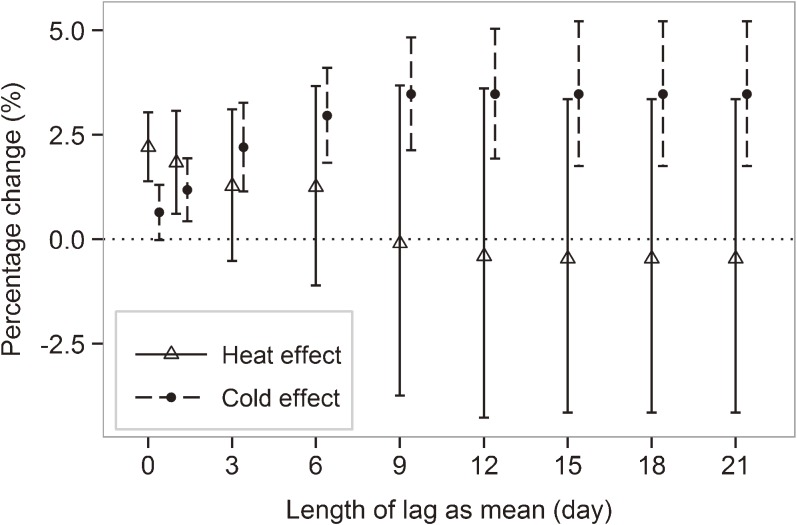
Combined percentage change in daily mortality due to heat and cold according to temperature lag. Estimates were based on relative changes in temperature. Lags, if any, were constrained as mean. Vertical lines denote the 95% CIs.

Significant increases in mortality risk due to exposure to heat and cold were observed (Table 3). In the fixed-lag models, no temperature lag was included for the estimation of the effects of heat, whereas lags up to 15 days (constrained as average) were included for all cities in estimating the effects of cold. In the second approach, lag duration was specified based on the lag structure for each city. For the effect of heat, an additional lag 1 was included for Tokyo; for the effect of cold, additional lags of 1 to 5 were added for Sapporo, lags 1 to 10 for Sendai and Osaka, and lags 1 to 15 for Tokyo, Nagoya, and Fukuoka. The increase in the risk of cold-related mortality was larger. Estimates were comparable between these lag selection strategies. After adjusting for O_3_ and PM_2.5_, the results did not vary much for cold, although the estimates for heat effect were slightly lower. In addition, estimates did not vary in relation to the *df* for the smoothing of the lag term (not shown). The stability of combined multicity effects against the length of delayed temperature effects was examined by gradually increasing the number of lags up to 21 days (Figure 3). Heat-related mortality risk was highest on the same day; the increase in the risk of cold-related mortality leveled out after approximately lag 9. Combined RR estimates were rather sensitive to the *df* of the temperature spline (eFigure). Greater *df* increased effect estimates even when no lag was specified. When the *df* were increased from 3 to 7, the cumulative effect of heat continued to increase for up to 1 week (6 lags). Therefore, all temperature splines were based on 3 *df* in this study.

**Table 3.** Combined percentage change in daily mortality due to heat and cold temperature, according to temperature lag specification and adjustment for air pollutants

Temperature lag and adjustment for air pollutant^a^	Heat effect^b^ (%)		Cold effect^c^ (%)	
	
	Estimate	95% CI	Estimate	95% CI
Fixed lag^d^				
Temperature only	2.21	(1.38–3.04)	3.47	(1.75–5.21)
With adjustment for O_3_	2.13	(1.30–2.96)	3.38	(1.66–5.12)
With adjustment for O_3_ and PM_2.5_	2.03	(1.16–2.91)	3.49	(1.74–5.27)

City-specific lag^e^				
Temperature only	2.21	(1.26–3.17)	3.57	(2.00–5.17)
With adjustment for O_3_	2.15	(1.26–3.04)	3.50	(1.93–5.10)
With adjustment for O_3_ and PM_2.5_	2.05	(1.14–2.97)	3.63	(2.03–5.26)

O_3_, ozone; PM_2.5_, particulate matter with an aerodynamic diameter of <2.5 µm.

^a^All models included confounding adjustment for season and longer time trend, day-of-the-week effect, and flu epidemics. Air pollutants were adjusted using the 3-day mean (lag 0–2).

^b^Estimates were computed by comparing mortality risks at the 99th and 90th percentile.

^c^Estimates were computed by comparing mortality risks at the 1st and 10th percentile.

^d^For models with fixed lag, no temperature lag was specified for all cities in estimating heat effect, while lags up to 15 days were allowed for all cities in estimating cold effect.

^e^For models with city-specific lag, a 1-day lag was specified for Tokyo and no lag for other cities in estimating heat effect. For cold effect, the lag interval varied by city: Sapporo with 5-day lags; Sendai and Osaka with 10-day lags; Tokyo, Nagoya, and Fukuoka with 15-day lags. Lags longer than 1 day were constrained as mean.

In all cities the increase in temperature-related mortality risk appeared to be unrelated to latitude, prevalence of AC, and unemployment rate (Table 4). Number of registered nurses was inversely related to the city effects of heat. The effect of heat was positively associated with average income per capita, cost of property rental, and population density.

**Table 4.** Percentage change in the city-specific effects of heat and cold temperature for a unit increase in selected community characteristics

Community characteristic	Change in heat effect^a^ (%)		Change in cold effect^a^ (%)	
	
	Fixed lag	City-specific lag	Fixed lag	City-specific lag
Latitude (°N)	−0.114	−0.119	−0.010	0.010
Air conditioner prevalence (%)	0.010	0.010	0.005	0.001
Population density (1000/km^2^)	0.011^c^	0.013^d^	0.005	0.003
Income per capita (100 000 Yen)	0.059	0.078^c^	0.042	0.021
Rental cost (1000 Yen/month)	0.048	0.062^c^	0.027	0.014
Registered nurses^b^	−0.102^c^	−0.112^d^	−0.077	−0.032
Unemployment (%)	0.032	0.017	−0.085	−0.033

^a^Percentage changes were estimated using bivariate meta-regression. Refer to the footnotes of Table 3 for a description of the methods used to select fixed and city-specific temperature lags.

^b^Unit is in standard score.

^c^*P* < 0.10.

^d^*P* < 0.05.

## DISCUSSION

Our findings indicate that the short-term mortality effects of heat and cold vary between northern Japan and the rest of the country. The effect of heat was generally immediate, except in the north (where it was negligible, likely because of the cooler climate). In Tokyo we noted a significant previous-day effect of heat. This 2-day effect is likely associated with dense buildup, which can lead to greater retention of heat and higher night-time temperatures.^10^^,^^12^ The findings of a study in Europe also suggested that urban mortality was more sensitive to heat.^44^ The heat effect at 1 to 2 days in Japan is consistent with previous findings from the United States,^2^^,^^45^ Canada,^46^ Europe,^6^ Mexico,^47^ Hong Kong,^48^ and Australia.^49^ A weak “harvesting effect” on mortality was noted in Osaka and Fukuoka. Harvesting effect refers to the hastening of imminent deaths after extreme weather events, such as heat waves or cold spells.^2^^,^^6^^,^^50^ In these 2 cities, heat exposure appeared to have shifted daily mortality forward, causing a temporary decrease in susceptible individuals on subsequent days. Because there are fewer susceptible individuals on subsequent days, inverse associations (RR < 1) may be observed over the few days immediately after a large positive heat effect. A short-term harvesting effect was less apparent in Sapporo and Nagoya and was not observed in Sendai and Tokyo. The absence of such an effect in Tokyo despite the significant effect of heat in the first 2 days suggests heat harmed the general population of the city, not just those close to death.^6^

The effect of cold was slower and longer, reflecting the presence of longer, indirect pathways, such as flu infection and other respiratory diseases, during winter.^51^ A study of 107 communities in the United States revealed a delayed effect of cold weather of up to 25 days^38^; the effect persisted up to 23 days in a study of 15 European cities.^5^ However, the effect was shorter (about 1 week) in a study of Montreal, Canada.^46^ In Japan, the duration of the effect of cold was short and varied geographically. The effect of cold persisted for multiple days up to approximately 1 week in all cities except Sapporo and Sendai, in the north. Delayed effects were especially noticeable in big cities with warmer climates such as Tokyo, Nagoya, and Osaka; Tokyo had lags up to approximately day 9. Studies in the United States and Europe also noted a larger cold effect in warmer cities.^2^^,^^3^^,^^5^^,^^52^^–^^54^ Cooler cities in northern regions showed almost no significant delayed effect of cold, suggesting that such communities were somewhat better adapted to cold weather. In some cities, the same-day (lag 0) negative effects of cold were mostly due to our modeling approach, which compared mortality risk at the city-specific minimum temperature to the 80th-percentile reference. This approach allowed us to examine the lag structure of temperature effect on extremely cold days, even though such minimum temperatures occurred on very few days. Another possible explanation is that human intervention resulting from early forecasting of extreme weather blunts the initial impact of cold. The overall increase in mortality due to cold was higher than the increase due to heat, reflecting the higher mortality observed during winter.^2^^,^^5^^,^^11^ Our combined estimates of the effects of heat and cold were comparable to those of similar studies of 107 US communities^38^ and London.^6^

Acclimatization to local climate is a result of physiologic changes,^55^ behavior modification, and changes in housing characteristics.^10^ To understand community acclimatization we compared city-specific effects estimated based on relative and absolute changes in temperature.^38^ As opposed to the relative effects, the absolute effects of temperature were more heterogeneous across cities, although not substantially so. This suggests a degree of acclimatization, since the same magnitude of temperature change resulted in impacts that differed by location. Communities with lower long-term temperatures, such as Sapporo, appeared less influenced by cold weather, as suggested by the lack of a delayed cold effect. The reverse magnitude for the relative and absolute effects of cold in Sapporo suggests that even though populations in cooler areas acclimatize to some extent, and thus are less affected by the same temperature drop as compared with those living in warmer climates, they are still adversely affected by the extreme coldness distinct to their climatic region (as evidenced by the positive, albeit nonsignificant, relative cold effect).

Tokyo, Nagoya, and Osaka—the 3 largest cities in the study—exhibited substantial heat-related mortality despite having higher long-term temperatures. Our findings suggest that populations in central Japan did not adapt well to warm climates and remained susceptible to heat. Mortality in these big cities was also vulnerable to displacement by severe cold weather. Comparable findings have been reported for other urban communities with temperate climates.^1^ The U-shaped temperature–mortality association observed in these cities has been reported elsewhere.^1^^,^^19^^,^^56^^–^^58^ As most deaths (>80%) were among adults older than 64 years of age, this study highlights the weak tolerance of urban elderly adults to extreme weather conditions. Susceptibility in large cities might also be related to the high prevalence of air conditioning units. Although it has been suggested that widespread use of heat-pump AC is linked to reduced physiologic acclimatization to weather (and therefore higher susceptibility to heat), current evidence is not conclusive.^10^

Despite regional variation, weather-related mortality risk in Japan did not appear to depend substantially on latitude. This contradicts previous findings in 11 US cities, which suggested a strong dependence of both heat and cold effects on latitude,^3^ although that study covered a wider range of latitudes (ie, about 23°N to 42°N). Prevalence of AC did not appear to influence variation in temperature effects across communities, in contrast to several studies that suggested an otherwise protective effect during summer.^38^^,^^43^^,^^59^ Consistent with previous reports,^12^^,^^13^^,^^41^ our findings show that large communities with high population densities, income per capita, and rents have a greater propensity toward increased risk of heat-related mortality displacement. The lack of nurses in large cities was also associated with greater mortality due to heat in our study. These findings underscore the importance of better understanding socioeconomic disparities in heat-related premature mortality in large cities, especially among elderly adults.^60^

The lack of a significant socioeconomic association, particularly for the city effects of low temperature, might be due to the use of prefecture-level data, as previously noted.^38^ Although major cities were included in this study, socioeconomic data at the prefecture-level might not fully represent characteristics at the city level, due to differences in geographic coverage. Studies using city- or individual-level data and longer time periods might yield better effect estimates. Our findings are further limited by the mismatch of time frame for certain socioeconomic variables, given the limited reports. The use of monitoring data from fixed stations as a surrogate for personal exposure also limits the interpretation of our results because individual exposure level can differ indoors and vary according to personal mobility and susceptibility. Our approach of using city-specific percentile cut-offs to quantify the effects of extreme temperatures did not account for the frequency and duration of extreme events. Further research is required to understand these added effects on premature mortality.^39^

In conclusion, short-term mortality displacement due to hot and cold weather varied according to regional climate in Japan. The densely populated cities in central Japan are particularly prone to the mortality burden of extreme weather. Northern communities are less affected, likely because of acclimatization and the generally cooler climate. Given the larger effect of cold weather, current prevention efforts, such as educational programs and early advisory system, should be extended to prevent cold-related premature mortality. Attempts to address weather-related health effects in countries with varying climatic regions should be community-specific, due to the heterogeneity of temperature effects across different locations.

## ONLINE ONLY MATERIALS

eFigure. Combined percentage change in daily mortality due to heat according to number of lags and degrees of freedom for the smoothing of temperature variable.

Abstract in Japanese.
