# Supplementary material for: Sociogeographic Variation in the Effects of Heat and Cold on Daily Mortality in Japan
Source: J Epidemiol. 2014 Jan 5;24(1):15–24. doi: 10.2188/jea.JE20130051 (PMC3872520; doi:10.2188/jea.JE20130051)
Supplement: eFigure. — Combined percentage change in daily mortality due to heat according to number of lags and degrees of freedom for the smoothing of temperature variable. [file je-24-015-s001.pdf]

Socio-geographic variation in the effects of heat and cold temperature on daily mortality in Japan

Chris Fook Sheng Ng*, Kayo Ueda, Ayano Takeuchi, Hiroshi Nitta, Shoko Konishi, Rinako Bagrowicz, Chiho Watanabe, Akinori Takami

*Corresponding author. E-mail address: chris.ng@nies.go.jp

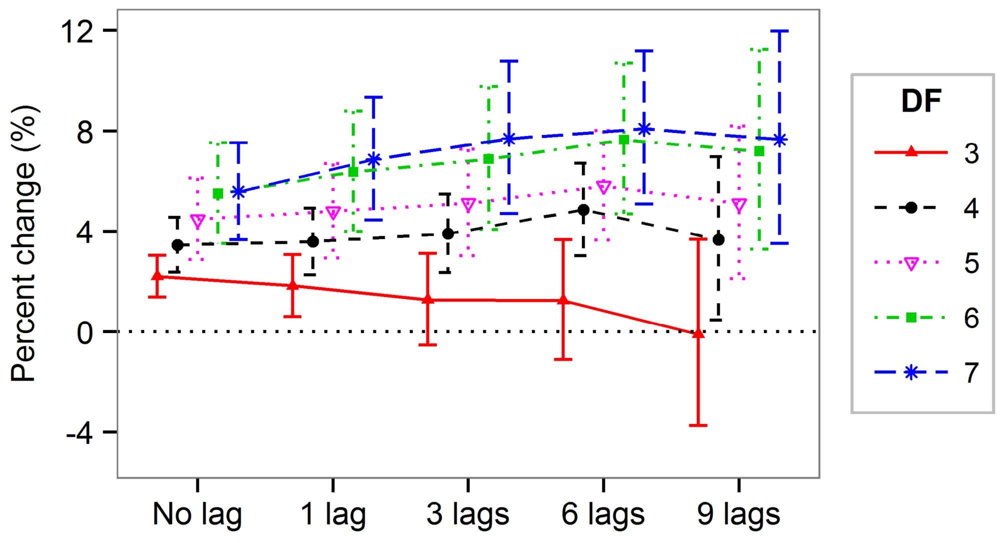

eFigure. Combined percentage change in daily mortality due to heat according to number of lags and degrees of freedom for the smoothing of temperature variable. Estimates were based on relative changes in temperature. Lags, if any, were constrained as mean. The figure shows changes in the estimates when the degrees of freedom (*df*) for the natural cubic spline (NS) of temperature variable were increased from 3 to 7 and the length of lags was increased from 0 to 9 days. The *df* for the NS of lag variable were fixed at 5. Vertical lines represent the 95% CIs. Except for the model with 3 *df* for temperature spline, all models with higher *df* showed an increasing heat effect as the number of lags gradually increased to 6 days. Even when no lag was included, the estimated heat effect increased when the *df* were increased from 3 to 7.
