# Supplementary material for: Sociogeographic Variation in the Effects of Heat and Cold on Daily Mortality in Japan
Source: J Epidemiol. 2014 Jan 5;24(1):15–24. doi: 10.2188/jea.JE20130051 (PMC3872520; doi:10.2188/jea.JE20130051)
Supplement: Abstract in Japanese. [file je-24-015-s002.pdf]

日本における高気温と低気温が日死亡に与える影響の社会地理学的変動

ウン・クリス・フック・シェン[1]，上田佳代[1]，竹内文乃[1]，新田裕史[1]，小西祥子[2,3]，バグロビッチ梨奈子[2]，渡辺知保[2]，高見昭憲[4]
1:国立環境研究所　環境健康研究センター　環境疫学研究室
2:東京大学大学院医学系研究科　人類生態学
3:ワシントン大学　人類学
4:国立環境研究所　地域環境研究センター

背景

外気温が感受性の高い集団の死亡に影響するということはよく知られているが，日本においてその関連の地域差は明らかにされていない．本研究では外気温と日死亡に対する影響の日本国内での変動を評価するための時系列解析を実施した．

手法

2002 年から 2007 年までの日本の 6 都市での死亡データ（事故死を除く）計 731,558 件を解析対象とした．外気温と日死亡との関連は，結果変数である日死亡数にポアソン分布を仮定した非線形分布ラグモデルで解析した．都市ごとに推定された結果は，都市を変量効果としたメタアナリシスで併合した．また，都市ごとの共変量は，都市を変量効果とした二変量メタ回帰によって調整した．

結果

高気温の影響は 1～2 日間継続し，暖かい地域での低気温の影響は 1 週間程度継続することが分かった．高気温によって上昇した併合死亡リスク(外気温 99%点でのリスクを 90%点でのリスクと比較して)は 2.21%増で 95%信頼区間は 1.38-3.04%，低気温によって上昇した併合死亡リスク(外気温 1%点でのリスクを 10%点でのリスクと比較して)は 3.47%増で 95%信頼区間は 1.75-5.21%であった．都市ごとの日死亡への影響は，外気温の絶対変化の影響の方が相対変化の影響よりばらつきが大きく，ある程度順応の影響が含まれている可能性が示された．涼しい気候の北部地域では低気温への順応がみられるが，極端に低い気温に対しては脆弱であることも示唆された．また，人口密度，平均収入，不動産賃貸料，看護師の数が都市間の外気温影響の変動に影響していることが示唆された．

結論

外気温関連死亡リスクの上昇には明らかな地域間変動が存在した．予防策を講じる場合には，地域間変動に影響する要因を検討する必要がある．
